# Progress testing 2.0: clinical skills meets necessary science

**DOI:** 10.3402/meo.v20.27769

**Published:** 2015-05-05

**Authors:** Jonathan Gold, Robin DeMuth, Brian Mavis, Dianne Wagner

**Affiliations:** 1 Department of Pediatrics and Human Development, Michigan State University College of Human Medicine, East Lansing, MI, USA; 2 Department of Family Medicine, Michigan State University College of Human Medicine, East Lansing, MI, USA; 3 Office of Medical Education Research and Development, Michigan State University College of Human Medicine, East Lansing, MI, USA; 4 Department of Medicine, Michigan State University College of Human Medicine, East Lansing, MI, USA; 5 Office of College-Wide Assessment, Michigan State University College of Human Medicine, East Lansing, MI, USA

**Keywords:** progress test, early clinical exposure, assessment, pilot, integration

## Abstract

**Introduction:**

Progress testing has been widely used in medical schools to test scientific knowledge but has not been reported for assessing clinical skills.

**Development:**

We designed a novel progress examination that included assessments of both clinical performance and underlying basic and social science knowledge. This Progress Clinical Skills Examination (PCSE) was given to 21 early medical students at the beginning and end of a 6-week pilot test of a new medical school curriculum.

**Implementation:**

This examination was feasible for early students, easy to map to curricular objectives, and easy to grade using a combination of assessment strategies.

**Future directions:**

Use of a PCSE is feasible for early medical students. As medical schools integrate clinical experience with underlying knowledge, this type of examination holds promise. Further data are needed to validate this examination as an accurate measure of clinical performance and knowledge.

Progress testing, with origins at the University of Maastricht, the Netherlands (1), and the University of Missouri Kansas City, is defined as ‘a longitudinal, comprehensive examination of knowledge acquisition and retention’ (2) and a ‘quality-controlled assessment tool for improving learning and teaching and the demonstration of educational standards’ (3). It has also been described as an assay of ‘functional knowledge’ (4). Progress tests sample the ‘complete knowledge domain’ (3) expected of graduating medical students and are delivered multiple times over the course of the educational program.

Because progress testing assesses the whole knowledge domain, it can be used for unique curricula with an unusual learning trajectory. The University of Maastricht needed a rational assessment strategy for its problem-based learning curriculum, one that would drive continuous, deep, learner-driven knowledge acquisition rather than test- or course-driven ‘binge learning’. The University of Missouri program, with entry into medical school directly from high school, required its assessment of students to take that uniqueness into account, and sought to demonstrate the rigor of their educational strategy. At this time, progress testing is taking place all over the world, and is described by a rich literature.

Advantages of progress testing described by that literature include the provision of integrated data for learners, faculty, and the overall curriculum over time (5); early prediction of students requiring remediation (2); and stability of the assessment process, as the same progress test can be administered as long as the same knowledge domain is desired, irrespective of the curricular strategy. Progress tests provide a ‘growth curve’ of knowledge that can illuminate curricular outcomes and support both learner and curriculum improvement. Albano et al. (6) demonstrated the differing ‘kinetics’ of six different curricula and also demonstrated little difference in final knowledge acquisition through the use of a common progress test sequence. Importantly, Schuwirth et al. (4) demonstrated that students experience less overall stress and anxiety when progress testing is employed as the major assessment strategy.

Progress testing has been applied to medical knowledge assessment but has not been described for clinical skills assessment. Nationally, medical educators are working to better integrate learning with actual performance through simulation or through work-based assessment. The movement toward entrustable professional activities (EPAs) (7) requires the assessment of integrated performance and drives the need for integrated assessment strategies.

We have embarked on a curriculum renewal process characterized by the integration of ‘necessary science’ (defined as the foundational biological, psychological, and social sciences needed to function as a new resident) and clinical skills throughout the entire medical education program. For many of the same reasons that medical knowledge progress testing was developed in the United States and the Netherlands, we needed to envision an assessment system that would support integration as the highest value for both our faculty and our students.

Toward this end, we created a ‘Progress Clinical Skills Examination (PCSE)’ requiring demonstration of integrated clinical skills and the necessary science knowledge underpinning those skills. We administered our PCSE twice as a pilot with early learners. Little has been published about the use of a clinical skills exam with early medical students, and the feasibility in this population was not clear. We describe the development, implementation, lessons learned, and the future directions of that PCSE.

## Development

### Setting

The student group for the PCSE was drawn from another pilot study, which included a 6-week trial of an early clinical experience (ECE). Our student sample was determined by the needs of this pilot. Of 21 students (9 male and 12 female), 7 had completed the first year of medical school, 9 had not yet matriculated, and 5 had completed a post-baccalaureate program. This pilot group was selected to represent a broad range of academic backgrounds and a range of previous clinical experience. All 21 students participated in the PCSE at the beginning and at the end of the ECE pilot test.

Our planned new curriculum is built on a framework of approximately 120 chief complaints and concerns (C3) topics, which define the competencies expected of our graduates. The end-competency template for each C3 is divided into three sections. The first section includes data gathering, problem identification and synthesis, and management. The second section includes the necessary science underpinning each clinical skill. The last section details the complexities and challenges we expect graduates to be able to identify and explore.

### Design of PCSE

The PSCE was designed as a multistation objective structured clinical examination (OSCE). In each station, students had 20 min with a standardized patient, family member, and/or healthcare team member, followed by 10 min to answer associated essay questions. The PCSE was blueprinted so that each station was linked to a specific C3 and each included assessments linked to all three parts of the end-competency template (see Table 1).

**Table 1:** Blueprint for PCSE

Chief complaints and concerns	Dysuria	Elevated blood pressure	Shortness of breath	Elevated temperature	Abdominal pain	Fatigue	Health maintenance	Diabetes
Communication skills challenges options	Embarrassing topic for teenagers	Parent and child in interview and physical exam	Worried patient	History from non-parent caregiver	Hard of hearing, blind, or demented	Reticent historian with hidden agenda	Complex and changing recommendations	Need for shared decision-making and non-judgmental stance
Data gathering: history components	Sexual history	Lifestyle, family history	Pulmonary, cardiac, hematologic compliance	Immunization history	Use of interpreter or family member	Depression or abuse or alcohol Multiple labs and tests on chart	Risk factors, patient goals, beliefs	Barriers to compliance
Data gathering: physical examination components	Pulse Pediatric Abdominal Exam (GU exam)	Vitals, including blood pressure, in both arms Lungs Heart Abdomen Extremities	Vitals Lungs Heart Vascular	Vitals Ears Lymph nodes Lungs Abdomen (meningeal signs)	Vitals Abdominal (rectal)	Vitals Lymph nodes Thyroid Lungs Heart Abdomen (breast/pelvic/ rectal)	Vitals Thyroid Lungs Heart Abdomen Vascular	Vitals Funduscopic Carotids Lungs Heart Abdomen Feet
Differential diagnosis of major active problem/s	By case scenario	By case scenario	By case scenario	By case scenario	By case scenario	By case scenario	By case scenario	By case scenario
Management plan	Antibiotic choice	Non-pharmacologic approaches	Medication changes necessary	In patient or outpatient work up	In patient or outpatient Imaging Antibiotics	Appropriate testing	Latest screening recommended	Lifestyle and pharmacologic approaches
Necessary science application options	Pathology Anatomy EBM Microbiology Pharmacology Ethics	Physiology Nutrition Genetics Public health	Anatomy Physiology Pharmacology	Micro Pharmacology Impact on family Safety	Anatomy Immuno Micro Pharm Path Nutrition	Patient impact Family impact Public health Ethics Psychopharm Behavior	Epidemiology Biostatistics Principles of screening	Biochemistry Nutrition Neurobiology of compliance/satisfaction
Controversies, Concerns, and complexities options	Stress Specter of abuse Confidentiality	Defining hypertension Addressing hypertension in a developmental context Treatment choices	Patient beliefs vs. biomedical etiology	Immunization evidence Parent and dependent interests	Antibiotics or not? Dietary recommendations	Chronic undifferentiated complaint Meds vs. talk therapy	Changing recommendation and challenging patient education	Control parameters Autonomy

Assessments for the PCSE included checklists completed by standardized patients and essay questions graded by faculty. We developed the essay questions to assess necessary science knowledge based on the relevant C3 end-competency template. The questions sampled a broad range of content, including physiology, anatomy, biochemistry, public health, safety science, ethics, pharmacology, and epidemiology. Grading rubrics for each essay question were developed by faculty using key concepts and/or key words.

We also received feedback from students about the PCSE as part of the pilot test evaluation.

The data from this project were reviewed by the Michigan State University Institutional Review Board and were determined to be exempt.

## Implementation

A four-station clinical skills exam was given to all students at the beginning of the 6-week pilot. Students interviewed and examined standardized patients and then answered three to six questions at a computer kiosk. The first exam occurred in a single morning. At the end of the pilot, the same four cases were used, along with four new cases. The number of necessary science questions answered was limited to three. The second exam occurred in a long afternoon.

The students were able to participate in the exam without difficulty, even though several had not started medical school and few had any clinical experience. All students completed the essay questions in the time allotted.

Three faculty members graded the essay portion of the PCSE. Using the rubrics, a single faculty member could grade all 21 students’ essays for a single station in less than an afternoon.

Students valued the experience; one student commented ‘The PCSE helped me get ready for the clinic. I got less nervous. It was like practice. The SP's were really good’. A faculty member commented ‘The surprising thing was that many students felt that the PCSE prepared them for clinic – an unintended consequence’. Although the PCSE was designed as an assessment of knowledge and skills, the exam was seen by students as another opportunity to practice for clinical care.

The delivery of the exam was different between the two points. Not only did the second iteration have more stations, it was also delivered after a multiple choice examination in the morning, and after a busy 6-week pilot. Given these limitations, comparison of student performance data has limited utility. However, students had no problems completing the longer PCSE in the time allotted.

## Future directions

As medical education moves toward a competency-based approach in which integrated clinical performance is the goal, the inclusion of a clinical skills component to progress examinations will be necessary to match assessments to curricular objectives. Our experience demonstrated that administration of the PCSE to early medical students is feasible. An important feature of this examination was the integration of the clinical encounter with essay questions to elaborate underlying necessary science, reflecting the integrated nature of the curriculum itself. Since this initial PCSE pilot test, we have successfully administered this examination to a sample of first through fourth year students at our institution to demonstrate its validity and its response to curricular effort. These administrations are providing baseline data as we transition to our new curriculum. As medical education moves to a more integrated approach to curriculum and assessment, this type of examination holds promise.
